# Transurethral Resection of the Prostate in Younger Men: Effectiveness and Long-term Outcomes

**DOI:** 10.5041/RMMJ.10520

**Published:** 2024-04-28

**Authors:** Kamil Malshy, Etan Eigner, Anna Ochsner, John Morgan, Ameer Nsair, Borivoj Golijanin, Michael Mullerad

**Affiliations:** 1 Department of Urology, Rambam Health Care Campus, Haifa, Israel; 2 The Minimally Invasive Urology Institute, The Miriam Hospital, Providence, RI, USA; 3 The Warren Alpert Medical School of Brown University, Providence, RI, USA

**Keywords:** Benign prostatic hyperplasia, effectiveness, outcomes, transurethral resection of the prostate, young patients

## Abstract

**Objectives:**

This study aims to investigate the efficacy and outcomes of transurethral resection of the prostate (TURP) in the context of younger male patients.

**Methods:**

Males aged ≤55 who underwent TURP at Rambam Health Care Campus from January 2011 to August 2023 were retrospectively reviewed. Clinicodemographic characteristics, indications for surgery, uroflowmetry, pressure-flow study, and early and late postoperative outcomes were collected. Patients with urethral or bladder abnormalities were excluded. Chi-square and Fisher’s exact tests were employed for bivariate analysis.

**Results:**

Inclusion criteria were met by 58 men who underwent TURP at a median age of 52 years (interquartile range [IQR] 49.5–54). Median prostate size was 35 mL (24.5–56), with median prostate-specific antigen of 1.4 ng/mL (0.65–3.1). A total of 60% of patients used α-blockers, and 19% used 5α-reductase inhibitors pre-surgery. Overall, 54 (93.1%) had severe lower urinary tract symptoms (LUTS), with 34 (59%) being predominantly emptying and 20 (35%) storage. Most surgeries were performed for refractory LUTS in 38 (66%), followed by urinary retention in 16 (28%). At 6 weeks, 57 (98%) patients were catheter-free. The maximum flow rate and residual volume showed significant improvement from a median of 6.85 mL/s to 17.9 mL/s (*P*<0.001), and from 120 mL to 10 mL (*P*=0.0142), respectively. Pathology revealed benign prostatic hyperplasia in 53 (91.4%), and inflammation in 5 (8.5%). A total of 13 auxiliary procedures were required in 12 patients (20.7%) during follow-up: 7 transurethral bladder neck incisions, 3 re-TURP, 1 meatus widening, and 1 patient required artificial urinary sphincter implantation followed by simple cystectomy for end-stage bladder.

**Conclusions:**

In young men, TURP showed short-term gains in flowmetry and catheter removal rates, but a sustained need for subsequent procedures in the long run. In this unique population, patients should be carefully selected, and alternative, less aggressive, interventions should be considered.

## INTRODUCTION

Benign prostatic hyperplasia (BPH) is one of the most prevalent urological conditions affecting men.^1^ This benign enlargement primarily occurs in the prostatic tissue, particularly the transitional zone, leading to a gradual narrowing of the urethral opening over the years.^2^ The result can be a wide variety of lower urinary tract symptoms (LUTS). These may be grouped into problems related to bladder emptying (e.g. weak stream, incomplete emptying) and storage (e.g. nocturia, urgency, and frequency).^3^ Although LUTS does not infer etiology, in men it is often due to BPH. In the United States, BPH/LUTS affects over 15 million men aged 30 and above,^4^ leading to a substantial economic burden of over US$3 billion per year.^5^,^6^

The prevalence of BPH is intricately tied to age, ranging from 8% in men in their 50s to nearly 80% in those aged 80 and above.^7^ Notably, the incidence of BPH/LUTS also varies according to age groups, with approximately 8% of men between 31 and 40 years affected, escalating to a staggering 90% prevalence among men aged 90 and above.^8^

Although the primary approach to managing bothersome LUTS related to BPH involves behavioral changes and medical interventions, predominantly with α-blockers and 5α-reductase inhibitors (5αRI), a considerable number of patients eventually proceed to surgical procedures. Indications for surgical intervention encompass refractory LUTS, recurrent urinary retention, urinary tract infections, refractory gross hematuria, obstructive uropathy, and the presence of bladder stones.^9^,^10^

For decades, transurethral resection of the prostate (TURP) has been a dominant surgical treatment for BPH.^11^ It is estimated that approximately 150,000 TURP procedures are performed annually in the United States.^12^ Despite numerous innovative alternatives, the impressive 90% success rate, high cost-effectiveness, and short learning curve have kept it as the first-line choice for prostates up to 80 mL in recent guidelines.^13^,^14^

Several factors contribute to the complexity of managing LUTS in younger patients, especially when considering surgical intervention. Firstly, bladder storage symptoms are approximately twice as prevalent as obstructive symptoms.^15^ Secondly, the etiology is less clear compared to older patients, in whom urethral stricture and bladder neck obstruction is more common than BPH, which causes intraluminal outlet obstruction.^16^ Thirdly, LUTS are often accompanied by pelvic and genital symptoms that are rarely solely attributed to bladder outlet obstruction.^17^,^18^ Lastly, the adverse effects of interventions at the prostate/bladder neck level may lead to ejaculation sequelae,^18^ which may carry more significance in younger individuals than in the elderly.

While the effectiveness and associated outcomes in older men are well-documented, the application of TURP in younger men remains less understood. This study aims to investigate the efficacy and outcomes of TURP in the context of younger male patients.

## MATERIALS AND METHODS

### Patients

This was a retrospective review of male patients aged 55 or younger who underwent elective TURP at Rambam Health Care Campus in Haifa, Israel, between January 2011 and August 2023. The TURP procedure, as defined by the ICD-9 code 60.29/60.2, specifies endoscopic resection of the prostate (Table 1). Therefore, to ensure that prostate resection rather than incision occurred, only cases with pathological tissue were included. In addition, patients presenting an endoscopic impression of bladder neck obstruction were considered eligible for study inclusion in cases where TURP was subsequently performed. Individuals with pre-existing urethral abnormalities, bladder abnormalities, or a history of prior interventions involving the bladder neck or prostate, or who had undergone emergency TURP procedures were excluded.

**Table 1:** ICD-9 Codes for Relevant Diagnoses and Procedures.

Condition/Procedure	ICD-9 Code
Benign prostatic hyperplasia	600.01
Transurethral resection of bladder neck	R57492
Transurethral prostatectomy	60.2
Other transurethral prostatectomy	60.29
Transurethral incision of prostate	60

### Study Design

This study was performed with institutional review board approval (protocol number RMB-0254-16). Data were extracted from electronic medical records maintained by our institution. The collected data encompassed patient demographics, preoperative clinical data including prostate volume (PV) (determined by transabdominal ultrasonography), prostate-specific antigen, LUTS assessment (further subdivided into predominant bladder emptying versus storage), medications (α-blocker, 5αRI, anti-muscarinic), uroflowmetry results, and indications for surgery. Additionally, the study incorporated pressure flow study findings, if performed. Male standard indexes were utilized for urodynamic definitions, including detrusor overactivity, characterized by detrusor contractions during the filling phase that may be spontaneous or provoked. The Bladder Outlet Obstruction Index indicates outlet obstruction when the following condition is met: Pdet@Qmax – 2Qmax > 40 (Pdet, detrusor pressure; Qmax, maximal urinary flow). Detrusor underactivity is defined by: Pdet@Qmax + 5Qmax < 100.^19^ Of note, all urodynamic studies were conducted within our neuro-urology division by a dedicated team and interpreted by specialized urologists. Refractory LUTS are defined as the failure of medical therapy to sufficiently alleviate bothersome LUTS.^13^

### Procedure Outcomes

The primary outcomes of our study were achievement of a catheter-free status within a 4–8-week period and evaluating the dynamics in pre- versus post-TURP flowmetry parameters. Secondary outcomes included assessment of perioperative complications graded by the Clavien Dindo Classification for patient contentment (defined as contented versus discontented based on the first follow-up visit), and long-term outcomes, defined as events occurring 12 months post-surgery, including the need for additional procedures. Standard follow-up protocol post-elective TURP was as follows: initial visit at 4–6 weeks, followed by subsequent visits every 3 months in the first year, with potential for shorter intervals based on patient concerns.

### Statistical Analysis

Statistical analysis employed chi-square tests and *t*-tests, with statistical significance set at *P*<0.05. All statistical analyses were conducted using Microsoft Excel (Microsoft Corporation [2021]).

## RESULTS

Throughout the study period, a total of 58 men met inclusion criteria, with a median age of 52 years (interquartile range [IQR] 49.5–54). The median PV was 35 mL (IQR 24.5–56), and the prostate-specific antigen level was 1.4 ng/mL (IQR 0.65–3.1). Among the participants, 35 (60%) were using α-blockers chronically, and 11 (19%) were on 5α-reductase inhibitors before surgery. Additionally, 2 patients (3.5%) used anti-muscarinic medications. Impressions of endoscopic bladder neck obstruction were observed in 8 (13.8%) patients. A summary of patient characteristics and preoperative parameters is provided in Table 2.

**Table 2:** Patient Characteristics and Preoperative Parameters in the TURP Cohort.

Parameter	Value
Number of patients	58

Age (y), median (IQR)	52 (49.5–54)

Min, max	34, 55

Prostate volume (mL), median (IQR)	35 (24.5–56)

PSA (ng/mL), median (IQR)	1.4 (0.65–3.1)
Pre-surgery medical treatment, *n* (%)	39 (67)
α-Blocker	35 (60.3)
5α-Reductase inhibitor	11 (18.9)
Anti-muscarinic	2 (3.4)

Patient comorbidities based on CCI, *n* (%)	
CCI: 0–2	46 (79.3)
CCI: 3–4	10 (17.2)
CCI: ≥5	2 (3.4)

Preoperative urinary retention status, *n* (%)	16 (27.6)
Permanent indwelling catheter	12 (20.7)
Clean intermittent catheterization	4 (6.9)
Number of preoperative trial of void attempts, median (IQR)	1 (0–2)
Predominant LUTS, *n* (%)	
Emptying	34 (58.6)
Storage	20 (34.5)

Pre-TURP flowmetry parameters, median (IQR)	
Voided volume (mL)	224 (179–345)
Qmax (mL/s)	6.85 (6–10)
Residual volume (mL)	120 (70–170)

Pressure flow study, *n* (%)	36 (62)
Obstructed	24 (41.3)
Detrusor overactivity	2 (3.44)
Detrusor underactivity	3 (5.2)
Stress urinary incontinence	1 (1.7)

CCI, Charlson Comorbidity Index; IQR, interquartile range; LUTS, lower urinary tract symptoms; max, maximum; min, minimum; PSA, prostate-specific antigen; Qmax, maximal flow rate; TURP, transurethral resection of the prostate.

### Preoperative Baseline Characteristics

In total, 54 men (93.1%) experienced significant LUTS, with 34 (59%) primarily characterized as emptying and 20 (35%) as predominantly storage difficulties.

**Figure 1:**
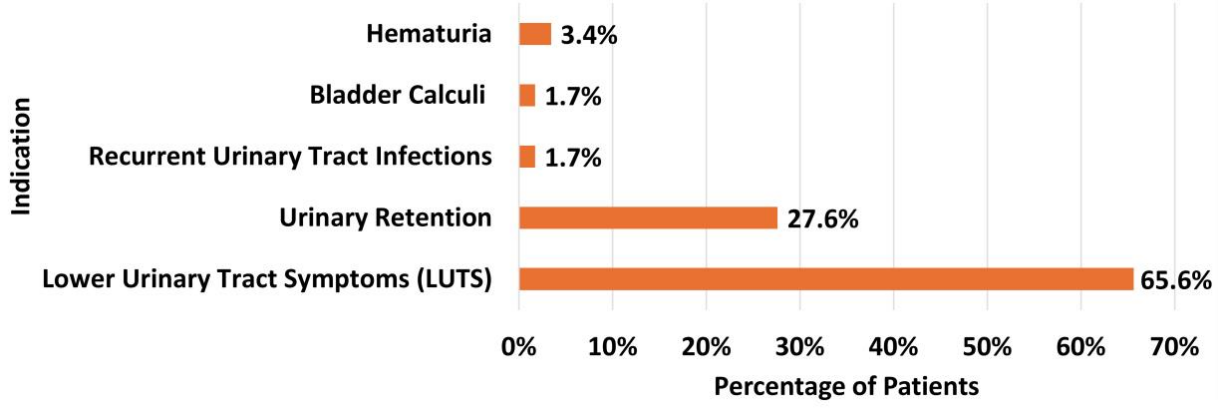
Indications for Transurethral Resection of the Prostate in Young Patients.

The surgeries were primarily performed to address refractory LUTS in 38 cases (66%). Additionally, urinary retention was the indication in 16 cases (28%). Other indications comprised hematuria in 2 patients (3.4%), bladder calculi in 1 patient (1.7%), and recurrent urinary tract infections in 1 patient (1.7%). Figure 1 provides a detailed breakdown of surgical indications for TURP. Preoperative flowmetry findings indicated a median voided volume of 224 mL (IQR 179–345), a median maximum flow rate of 6.85 mL/s (IQR 6–10), and a residual volume of 120 mL (IQR 70–170).

A pressure flow study was conducted in 36 patients (62%), revealing Bladder Outlet Obstruction Index within the obstructed spectrum in 29 (81%) cases and detrusor underactivity in 3 (8.3%).

### Perioperative Outcomes

The mean postoperative length of stay was 2.4 days (standard deviation 0.76). The median duration with a catheter before removal was 2 days (range 1–10). Notably, 5 out of 58 patients (8.6%) were discharged home with a catheter due to failure of the first void trial. In total, 3 patients (5.2%) experienced perioperative grade 2 Clavien Dindo Classification, of whom 2 developed febrile urinary tract infections, and 1 required a blood transfusion due to hematuria. No higher-grade complications were observed. Notably, during the postoperative period, only 1 (1.7%) out of 58 patients failed the trial without a catheter.

### Short-term Outcomes

The initial postoperative office visit occurred at a median of 5.4 weeks (IQR 4.6–7.4), and catheter-free status was observed in 57 (98%) patients. The maximum flow rate and residual volume significantly improved from a median of 6.85 mL/s to 17.9 mL/s (*P*<0.001), and from 120 mL to 10 mL (*P*=0.0142), respectively (Table 3). Of the 58 patients, 11 (19%) subjectively expressed short-term discontentment related to erectile dysfunction (*n*=5; 9%), urinary incontinence (*n*=6; 10%), dysuria/pelvic pain (*n*=5; 9%), and retrograde ejaculation (*n*=2; 3.5%). Pathology revealed BPH in 53 (91.4%), and inflammatory changes in 5 (8.5%). Prostate cancer was not detected in any case.

**Table 3:** Pre- versus Post-TURP Flowmetry Parameters.

Flowmetry Parameter	Value median (IQR)	*P* Value
Voided volume (mL)		
Preoperative	224 (179–345)	0.89
Postoperative	228 (134–286)	

Qmax (mL/s)		
Preoperative	6.85 (6–10)	<0.001
Postoperative	17.9 (11–31)	

Residual volume (mL)		
Preoperative	120 (70–170)	0.0142
Postoperative	10 (0–60)	

Qmax, maximum flow rate.

### Late Results

**Figure 2:**
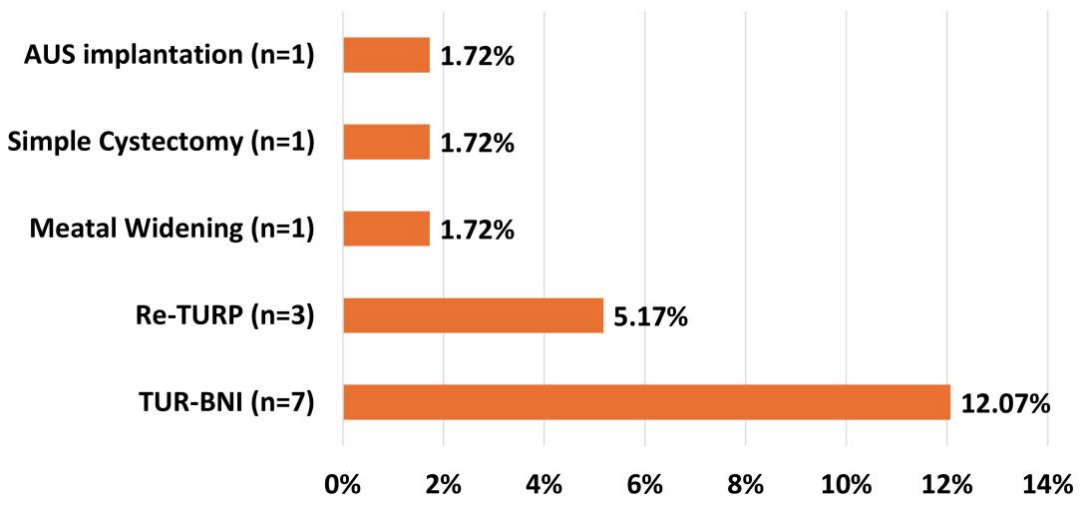
Long-term Auxiliary Procedures Performed in 12 Patients. A total of 12 patients required 13 auxiliary procedures. AUS, artificial urinary sphincter; re-TURP, repeat transurethral resection of the prostate; TUR-BNI, transurethral bladder neck incision.

Of the 58 patients studied, 23 (39.7%) were lost to follow-up after their first postoperative visit, resulting in a median overall follow-up time of 42 days (range 17 days–8.8 years). Overall, 18 patients (31%) reported persistent, recurring, or worsening LUTS. Within this follow-up timeframe, 12 patients (20.7%) required repeat or auxiliary procedures (Figure 2). Only 1 of the 8 patients who had bladder neck obstruction (BNO) pre-TURP needed subsequent transurethral bladder neck incision (TUR-BNI).

## DISCUSSION

This retrospective study sought to investigate the short- and long-term TURP outcomes in young men. Our results demonstrated significant improvement in all uroflowmetry parameters. Moreover, the high rates of patients discharged without a catheter and those maintaining catheter-free status during the initial follow-up visit support the notion that an obstructive element was effectively addressed. This observation becomes particularly evident when considering that almost two-thirds of our cohort experienced predominant emptying symptoms, and over 25% underwent the procedure due to refractory urinary retention.

While extensive data on TURP outcomes and adverse events are available, there is a notable gap in research focusing on young patients, particularly concerning the safety and feasibility in this demographic. Del Rosso et al. conducted a prospective study with a cohort of 18 patients aged 50 and below (median PV of 36 mL) undergoing modified TURP to mitigate retrograde ejaculation development chances.^20^ Their findings demonstrated a substantial increase in maximum flow rate from 7.4 mL/s preoperatively to 23.6 mL/s during follow-up, aligning with our own results. Furthermore, the modified mini-invasive TURP, which involves resection of the bladder neck at the 6 o’clock position, followed by a resection at the 12 o’clock position, was demonstrated to maintain patients’ baseline International Index of Erectile Function and Male Sexual Health Questionnaire scores after 1 year of follow-up.^20^ However, it should be noted that our retrospective study design limited our ability to obtain objective measurements on long-term sexual functions.

Retrograde ejaculation, the infamous adverse effect of TURP, is the primary reason for the shift toward less aggressive resections. Transurethral *incision* of the prostate is the preferred alternative in cases were PV is <30 mL.^13^,^14^ This procedure has been shown to have a significantly lower rate of retrograde ejaculation (22.5%) and erectile dysfunction (7.5%) compared to TURP (52.5% and 20%, respectively).^21^ Despite the appealing advantages of this technique, studies have shown that it is under-utilized.^22^ Given the median prostate size of 35 mL and guidelines recommending restriction of this procedure for prostates <30 mL, at least half of our cohort would have been suitable for alternative options.

Starting in 2004, the non-thermal prostatic urethral lift (PUL; UroLift®, NeoTract Inc., Pleasanton, CA, USA) has been seen as a safe alternative, offering the benefit of avoiding ejaculation-related issues in prostates smaller than 70 mL.^23^ To assess UroLift’s safety in young patients, Annese et al. studied the results of 35 patients with an average age of 50 (standard deviation 11), including 18 individuals under the age of 50.^24^ After a follow-up period of over 12 months, there was a significant reduction in International Prostate Symptom Score and post-void residual volume (20 to 11 points; 70 to 22.5 mL, respectively), along with a notable 68% improvement in the maximum flow rate.^24^

While there was no specific breakdown into age groups, PV <45 mL was identified as a positive indicator. Importantly, the International Index of Erectile Function–5 and Male Sexual Health Questionnaire–Ejaculatory Dysfunction–Short Form (MSHQ-EjD-SF) scores remained consistent throughout at least 12 months of follow-up.^24^

Despite the improvements observed in short-term clinical and flowmetry parameters, one-third of our cohort reported persistent, recurring, or worsening LUTS during follow-up. Moreover, over 20% required subsequent or additional procedures, primarily involving TUR-BNI. This less favorable outcome could be theoretically attributed to an underlying BNO as the primary cause of emptying symptoms, rather than BPH, with obstruction relapsing within a relatively short period. Interestingly, a minority of patients requiring auxiliary TUR-BNI had a pre-TURP indication of bladder neck involvement.

It is widely suggested that, for younger men experiencing obstructive voiding symptoms without urethral stricture disease, a comprehensive evaluation for primary BNO is essential and should be considered for exclusion. In a recent cohort of more than 1,200 patients experiencing LUTS, 11% were identified with BNO, notably within the younger subgroup.^16^

The formation of fibrotic strictures post-TURP may be a secondary cause for this sequela. Complications, including urethral and bladder neck stricture, may occur in around 9% of patients following TURP. Prolonged surgical duration and elevated prostatic volume are specifically recognized as the most relevant risk factors in reported cases.^25^ Currently, there is no clear advantage of bipolar TURP over monopolar TURP in preventing bladder neck *contracture*.^26^

To the best of our knowledge, this represents the largest documented cohort that examines both short- and long-term outcomes of TURP in this distinctive population. The notably unexpected prevalence of bladder neck contracture, necessitating TUR-BNI in over one-fifth of the patients, is a significant and valuable addition to the existing literature on this procedure.

## LIMITATIONS

This study had several limitations. Firstly, being a single-center study might limit the generalizability of our findings. The outcomes may not fully encapsulate the diverse population and practices observed in alternative healthcare settings. An example within our cohort includes the undistinguished utilization of both monopolar and bipolar TURP techniques. It should be noted that TURP syndrome remains a major concern. Triggered by excessive absorption of glycine (the electrolyte-free irrigation fluid utilized only in the monopolar energy system^11^), this syndrome can significantly impact patients’ length of stays. With that said, TURP syndrome occurrence has dwindled in recent years, with incidence rates ranging between 0.78% and 1.4%.^27^ Current urological guidelines advocate for various aspects of this surgical technique, including the utilization of both bipolar and monopolar TURP without distinction.^13^,^14^

Secondly, this study performed only a limited assessment of long-term sexual function, since objective measurements were not available. The scarcity of detailed information regarding sexual outcomes could hinder a comprehensive understanding of the influence of TURP on the sexual health of younger patients.

Thirdly, the retrospective nature of our study, spanning more than 10 years, limited the consistent utilization of acceptable questionnaires for quantifying LUTS (e.g. International Prostate Symptom Score) both pre- and post-procedure. However, it is important to note that a thorough history regarding storage and emptying symptoms was described in our results.

Fourthly, we observed high lost-to-follow-up rates within our outpatient clinic due to the tendency of patients to seek follow-up elsewhere within the healthcare system. Hence, theoretically, additional patients could have experienced late sequelae of which we were unaware.

Lastly, since the focus of this study was investigation of TURP results, alternative procedures were underpresented, and their utilization was not extensively explored. This restriction could affect the inclusivity of the discussed treatment options, especially in terms of their extensive application within this specific age category.

## CONCLUSIONS

This was a retrospective cohort study that examined the outcomes of TURP in young patients. The study presented conflicting outcomes, with notable short-term improvements in flowmetry parameters and catheter removal rates, but substantial requirement for subsequent procedures in the long term. This highlights the critical significance of careful patient selection within this distinct population and emphasizes the availability of alternative, less aggressive interventions within the urologist’s arsenal.

## Abbreviations

BNO: bladder neck obstruction
BPH: benign prostatic hyperplasia
IQR: interquartile range
LUTS: lower urinary tract symptoms
PV: prostate volume
Pdet: detrusor pressure
re-TURP: repeat transurethral resection of the prostate
TUR-BNI(s): transurethral bladder neck incision(s)
TURP: transurethral resection of the prostate
